# Reviews and Book Notices

**Published:** 1880-11

**Authors:** 


### Reviews and Book Notices.

ARTICLE X.—A CONTRIBUTION TO A KNOWLEDGE OF FRACTURE OF THE RIM OF THE ACETABULUM, BASED ON THE REPORTS OF TWENTY-SEVEN CASES AND EXPERIMENTS ON THE CADAVER. By N. Senn, M.D., Milwaukee.

This is a pamphlet of thirty pages, neatly reprinted from the Transactions of the State Medical Society of Wisconsin.

The author of this paper, which was read to the Wisconsin State Medical Society, commences with the following proposition: "Fracture of the rim of the acetabulum is an extremely rare accident, being less frequent than fractures involving the floor of the acetabulum."

After giving a concise and interesting description of the anatomy of the acetabulum, he details seven experiments on five different cadavers, instituted for the purpose of determining the effect of blows applied either to the knee or foot, with the limb in such direction that the force of the blow would be sustained by some part of the rim of the acetabulum. Concerning the results of these experiments, Dr. Senn remarks: "These experiments demonstrate how difficult it is to fracture the rim of the acetabulum, and they tend to corroborate the views of the majority of surgeons, who consider this injury one of the rarest accidents in surgery. They are also instructive in showing where bones are most likely to break, when a sufficient amount of violence is brought to bear upon certain parts of the skeleton. Thus, although in every experiment the limb was placed in the most favorable position, and the force applied in such a manner as to expend itself on the inner surface of the acetabulum, fracture of the rim was produced only once, and then by forcibly rotating the femur inwards, and by applying the force to the posterior margin of the trochanter." By a commendable degree of research through surgical literature, from the days of Sir Astley Cooper to the present time, the author collates the history of twenty-six cases of this variety of fracture, to which he adds an interesting one occurring in his own practice. After discussing the causes, symptoms and diagnosis of this rare variety of fracture, giving special attention to the differential diagnosis between it and fracture of the neck of the femur without impaction, he says: "Crepitus and a tendency to reluxation are the symptoms on which we place most reliance to differentiate this fracture from simple dislocation. The akystopeurastic of Middeldorpf may be of great service to determine the existence of fracture of the rim. After reduction has been accomplished, a long, stout needle is passed through the tissue to the supposed seat of fracture. By lateral movements of its point, the defect in the margin, as well as the roughness of its surface, is ascertained. An effort should now be made to fix the detached fragment with the point of the needle, and, by rubbing it over the broken margin, a rough crepitus is elicited."

In regard to the prognosis, Dr. Senn says:

"The prognosis must have reference to the preservation of life and the restoration of the utility of the limb. All of the old authors regarded fracture of the pelvic bones as a grave lesion, almost necessarily leading to a fatal termination. I believe with Dr. E. Andrews, that all uncomplicated fractures of these bones tend to recovery, and that death is attributable in most instances to a lesion of some important pelvic or abdominal viscera. In twenty-three cases where the result is noted in this regard, thirteen recovered, and ten died. The prognosis is less favorable if the floor of the autobulum is also implicated in the fracture. Of four cases of this sort, only one recovered. In nine cases out of the thirteen that recovered, the limb remained in place after reduction, and the recovery was complete. In four cases redislocation took place, the limb assuming the same malposition as after simple, unreduced dislocation of the femur."

The whole paper is worthy of careful perusal, and does credit to its industrious author. We quote what he says concerning the *treatment* of fractures of the acetabulum entire, as follows:

TREATMENT.

The indications to be fulfilled in the treatment of this class of injuries are: 1st. To reduce the dislocation; 2. To retain the head of the femur in the socket until union has taken place between the fragments.

The dislocation may be reduced by manipulation or by extension; in both instances flexion constitutes an important step in the operation. Bigelow says:* "These displacements, especially the displacement backwards, demand the usual attempts at reduction by flexion. Although the bone inclines to slip from the socket, it can be retained there, in cases of a sort heretofore considered difficult of treatment, by angular extension, with an angular splint attached to the ceiling, or some other point above the patient; or if any maneuver has reduced the bone, the limb should be retained, if possible, in the attitude which completed the maneuver."

In seventeen of the cases reported, the manner of reduction is specified as follows: By extension, eleven—in most of these cases extension and flexion were combined; by manipulation, two; by manipulation and extension, one; by manipulation over Sutton's fulcrum, one; by extension with pulley, two. In all but one of the cases the displacement was corrected without difficulty. In Pooley's case it was supposed that the detached fragment prevented reduction by being placed between the head of the femur and the acetabulum. In my own case, the use of the pulley was required from the length of time that had elapsed since the injury had been received. Had I been aware of the nature of the injury, no attempt would have been made to reduce the dislocation, as a restoration of the actabulum could not be hoped for after such a long lapse of time since the injury was sustained. Permanent extension was applied more for the purpose of determining a favorable locality for the formation of a new joint, than with a view of retaining the head of the bone in its socket.

---

* Dislocation hip joint.

As in most instances a diagnosis cannot be made before reduction has been accomplished, surgeons will resort to their favorite methods of reduction. Should the nature of the lesion be determined beforehand, traction in the direction of the broken edge of the rim, and rotation of the limb inwards, will readily restore the normal relation of the parts. As we possess no direct measures of keeping the fractured surfaces in apposition, all our efforts must be directed toward preventing reluxation, by appropriate position and fixation of the limb and pelvis.

The depth and extent of the fractured margin, as well as the location of the fracture, will determine the difficulty in retaining the head of the femur in its normal position. If sufficient depth of the upper portion of the rim is left to serve as a support to the head of the bone, all that is necessary is to dress the thigh in the abducted position, so as to press the head of the femur against the floor of the acetabulum. As the contusions of the soft parts about the hips and pelvis are severe, a plaster-of-Paris splint cannot be applied as a primary dressing. The healthy limb and pelvis should always be included in the retentive dressing.

"Bonnet's wire breeches, Dzondi-Hagedorn's apparatus, or Hamilton's splint, as advised by him in the treatment of fractures of the femur in children, will be found sufficient to maintain retention.

"After the swelling in the soft parts has subsided, nothing more perfect could be devised than a plaster-of-Paris dressing, including both limbs and the pelvis.

"When nearly the entire depth of the upper or posterior portion of the rim has been detached, muscular contraction must be counteracted by permanent extension with the weight and pulley, and immobility of the joint secured by appropriate splints. In cases of this sort, angular extension with an angular splint, as advised by Bigelow, will answer an admirable purpose. The unbroken part of the rim should be made the support for the head of the femur whenever practicable, as when the posterior part of the rim is fractured, the thigh should be dressed in the position of hyper-extension, a broad, firm, pelvic band, with a compress above the trochanter, will aid in keeping the bone in place, in approximating the fractured surfaces, and in preventing muscular spasms.

"The treatment should be continued for a sufficient length of time to secure a firm union of the detached fragment with the broken rim, which, as in other fractures, generally requires from four to six weeks. The patient must be directed to exercise great care in the use of the limb for a considerable length of time after all dressings have been removed, so as to obviate any undue pressure against the recently repaired rim of the acetabulum.

ARTICLE XI.—THE HYPODERMIC INJECTION OF MORPHINE; ITS HISTORY, ADVANTAGES AND DANGERS. By H. H. Kane, M.D., New York. Published by Chas. L. Bermingham & Co. 1880.

In 1878 Dr. Kane commenced a careful study of his subject, and by personal inquiry, letters, and articles in journals, throughout this country, Great Britain, France and Germany, gathered a vast amount of material. From this large mass of information he has written a most valuable book of three hundred and fifty pages. Especially valuable will it be to the people of our country, if will serve as a note of warning to those who indirectly, although innocently in many cases; are the cause of this habit. From the facts set forth in chapters one and two, the author thinks he is justified in drawing the following conclusions: That localization of the injection is not necessary, and sometimes mischievous, save in certain cases, that rapidity of absorption varies in different individuals, and in the same individual at different times; that hypodermic injections blunt sensibility sufficiently to admit of the performance of minor surgical operations without the use of an anæsthetic; that abscesses are comparatively rare; that to acid solutions, unclean syringes, rusty or dirty needles and solutions which have stood for some time unprotected by salicylic acid, carbolic acid or some such drug, are the causes which produce erysipelas, pyæmia and certain other accidents which follow the hypodermic procedure.

In chapter three, in which is discussed the commencing and the usual dose—idiosyncrasy, narcotism and elimination of morphia by the kidneys, certain experiments are cited which seem to demonstrate the fact that in dogs and rabbits, at least, interference with the renal function renders doses of morphine fatal that would otherwise cause only a stupor of some hours duration.

Among the causes which produce certain alarming symptoms, following immediately upon the injection subcutaneously of morphia, Dr. Kane speaks of *injection into a vein*, *alarming syncope* and *rapid absorption.* In the treatment of the last named accident he recommends very highly the tourniquet, or more properly a strap armed with a patent buckle. At the first intimation of danger after a hypodermic injection this should be applied above the puncture in the arm, and should be pulled tight and kept so for several hours. This being loosened gradually, but a small amount of the drug is permitted to enter the general circulation until danger is passed.

The author, in a chapter devoted to the subject, speaks at length of the treatment of opium narcosis. He tabulates all the remedies usually found of benefit in these cases as follows:

To aid to establish respiration.

To stimulate the heart.

To produce general stimulation.

To counteract soporific effects.

To produce diuresis.

He believes opium and belladonna mutually antidotal in certain particulars only, and cites the experiments and conclusions of many well known writers upon the subject. The value of coffee, or its active principle, is also discussed.

The question of using morphia with atropia is discussed in chapter 8. The conclusions of Bartholow, who has studied their physiological action very closely, are given, with our author's deductions, who says, in closing, "that there is considerable room for exact clinical inquiry as to the advantage of combining the two drugs for hypodermic use."

The dangers peculiar to, and treatment of the opium habit are discussed, and with the conclusions, end the book. In view of the rapid increase in the importation and sale of opium, the reckless prescribing of some of our profession, and the sad and terrible effects seen in scores of opium habituates, we regard the publication of this book as particularly opportune. While the writer of this brief review believes that the author rather overestimates the frequency of the use of the hypodermic syringe, his observation and experience fully confirm Dr. Kane's conclusions in regard to the baneful use of the different preparations of the day, and from recent investigations I am fully convinced that it is from our profession that the first dose is received. As a profession we are, I am afraid, careless in prescribing this drug, and unmindful of the best interests of our patients when we permit them to continue the use of a remedy which may produce such physical and moral disaster.

ARTICLE XII.—MINOR GYNÆCOLOGICAL OPERATIONS AND APPLIANCES; FOR THE USE OF STUDENTS. By J. Halliday Croom, M.B., M.R.C.P.E., F.R.C.S.E., Lecturer on Midwifery and the Diseases of Women at the School of Medicine; Physician Royal Maternity Hospital; late Tutor to the Midwifery Class, University, Edinburgh. E. & S. Livingstone, 57 South Bridge, Edinburgh; 1879.

If this book had been written for the use of practitioners, instead of for students, little harm would have been done. But as it is intended "for the use of students," there is a probability that under-graduates will get a wrong impression of gynæcology from a perusal of its pages. To a physician of experience, this book may be of some benefit, in that it will serve to freshen the memory on some forgotten points, but it will not give any great amount of knowledge on any subject which it treats.

A medical student ought not to read such a book as this, at least not until he has read several works on gynæcology which treat the subject exhaustively; for, if he gets his first ideas of the diagnosis and treatment of the diseases of women from a work of this scope, he will be apt to have a very superficial knowledge of the subject.

The book contains ten chapters, each one upon a very important subject; and yet the entire work contains but 102 pages, 12mo.

Had the author devoted all this space to the consideration of some one of the ten chapters, as "Cervical Applications," or "The use of the Sound," he would have given to the medical profession something of real benefit. As it is, the book is of very little value. "Hand-books" and "Manuals" of medicine and surgery are altogether too plentiful. Their tendency is towards superficial rather than thorough education.

Every physician who writes a monograph upon any topic, and embodies in his paper all that is known in regard to the subject he discusses; who, in other words, gives all the details as well as the main facts in the case, puts something into the hands of his readers that it is worth the having. It is comparatively an easy task to write a "hand-book," while to become the author of an exhaustive monograph, involves a great deal of study and research. B. W. G.

ARTICLE XIII.—ON REST AND PAIN. A Course of Lectures on the Employment of Mechanical and Physiological Rest in the Treatment of Accidents and Surgical Diseases, and the Diagnostic Value of Pain. By John Hilton, F.R.S., F.R.C.S., Professor of Anatomy and Surgery, etc., etc.

This book is already so well known to professional readers as to need little comment. It consists of a series of lectures in which Mr. Hilton shows how largely professional success in surgery is due to the recuperative powers of nature, especially when helped by the suggestions of the thoughtful surgeon, and that growth and repair bear an exact relation to physiological rest, local and general. Much consideration is given to pain, local, peripheral, sympathetic; numerous cuts show the peripheral distribution of the nerves of sensation. The work is of interest to the advanced student as well as the busy practitioner. It is the first of Wm. Wood & Co.'s phenomenally cheap library of standard medical authors.

ARTICLE XIV.—A TREATISE ON COMMON FORMS OF FUNCTIONAL NERVOUS DISEASES. By L. Patzil, M.D.

This author characterizes those diseases as functional, in which pathological anatomists can find no centric cause, and in which the changes are of a molecular nature. He considers that due attention is not paid to these affections in English text books devoted to nervous diseases, though practically he considers them more important by far than diseases of organic lesion and much more frequently encountered.

He takes up in successive detail, chorea, epilepsy and neuralgia in its various localities, omitting consideration of hysteria as already sufficiently described in works before the public. The remaining 186 pages are given to peripheral paralysis; some of the forms given, however, are not considered strictly functional, but are added for the sake of completeness of the subject in every detail. The subject is fully discussed in etiology and treatment, and well illustrated by cuts giving the nervous peripheral distribution and the motor points of muscles.

The work is interesting and clear in style, and is the fifth of William Wood's series for 1880. D.

ARTICLE XV.—A TREATISE ON THE DISEASES OF THE EYE. By J. Soelberg Wills, F.R.C.S., Director of Medicine of the University of Edinburgh; Professor of Ophthalmology in King's College, London; Ophthalmic Surgeon to King's College Hospital; and Surgeon to the Royal London Ophthalmic Hospital. Third American edition, from the third English edition, with copious additions by Charles Stedman Bull, A.M., M.D., Surgeon and Pathologist to the New York Eye and Ear Infirmary; Lecturer on Ophthalmology in the Bellevue Hospital Medical College. Philadelphia: Henry C. Lea's Son & Co.; 1880.

It is scarcely necsssary to do more than inform the readers of the JOURNAL AND EXAMINER that a new edition of this work has just appeared.

The facts that the original work met "with so very favorable a reception both by the profession at large and by the British and foreign medical press, and especially that it should have been deemed worthy of being translated into French and German," are indicative of its great merit.

The annotations of the editor, Dr. Bull, place before the student an excellent synopsis of the advances which have been made in ophthalmology during the past few years.

The treatise is illustrated with a large number of cuts, and is printed with clear type on good paper. E. L. H.

ARTICLE XVI.—NASO-PHARYNGEAL CATARRH. By Martin F. Coombs, M.D., Professor of Physiology, Opthalmalogy and Otology, in the Kentucky School of Medicine, etc., etc.

There is nothing very new in this work. As the author states in his preface, he may have satisfied the demands of a few friends who urged him to the task, and yet fallen far short of the requirements of a large class of practitioners. Though there is an interesting chapter on Infusorial Catarrh, the rest of the cases detailed, results of treatment, cuts of instruments used, etc., are such as are familiar to every practitioner.

